# Pneumocystis Pneumonia in HIV-Infected and Immunocompromised Non-HIV Infected Patients: A Retrospective Study of Two Centers in China

**DOI:** 10.1371/journal.pone.0101943

**Published:** 2014-07-16

**Authors:** Fei Guo, Yong Chen, Shuang-Li Yang, Huan Xia, Xing-Wang Li, Zhao-Hui Tong

**Affiliations:** 1 Department of Respiratory and Critical Care Medicine, Beijing Institute of Respiratory Medicine, Beijing Chaoyang Hospital, Capital Medical University, Beijing, Republic of China; 2 Center for Infectious Diseases, Beijing Ditan Hospital, Capital Medical University, Beijing, Republic of China; BarcelonaUniversity Hospital, Spain

## Abstract

**Background:**

Pneumocystis pneumonia (PCP) is an emerging infectious disease in immunocompromised hosts. However, the clinical characteristics of these patients are poorly understood in mainland China.

**Methods:**

We performed a retrospective study of PCP from 2008 to 2012. Information was collected regarding clinical manifestations, hospitalization, and outcome. A prognostic analysis was performed using a Cox regression model.

**Results:**

151 cases of PCP were included; 46 non-HIV and 105 HIV cases. All-cause mortality (15.2% vs. 12.4%, *p* = 0.64) and the results of time-to-event analysis (log-rank test, *p* = 0.62) were similar between non-HIV and HIV infected cases, respectively. From 2008 to 2012, time from admission to initial treatment in non-HIV infected PCP patients showed declining trend [median (range) 20 (9–44) vs. 12 (4–24) vs. 9 (2–23) vs. 7 (2–22) vs. 7 (1–14) days]. A similar trend was observed for all-cause mortality (33.3% vs. 20.0% vs.14.3% vs. 14.3% vs. 6.7%). Patients with four or more of the following clinical manifestations (cough, dyspnea, fever, chest pain, and weight loss) [adjusted HR (AHR) 29.06, 95% CI 2.13–396.36, *P* = 0.01] and admission to intensive care unit (ICU) [AHR 22.55, 95% CI 1.36–375.06, *P* = 0.03] were independently associated with all-cause mortality in non-HIV infected PCP patients. Variables associated with mortality in HIV infected PCP patients were admission to ICU (AHR 72.26, 95% CI 11.76–443.87, *P*<0.001) and albumin ≤30 g/L (AHR 9.93 95% CI 1.69–58.30, *P* = 0.01).

**Conclusions:**

Upon admission comprehensive clinical assessment including assessment of four or more clinical manifestations (cough, dyspnea, fever, chest pain, and weight loss) in non-HIV infected PCP patients and albumin ≤30 g/L in HIV infected patients might improve prognosis.

## Introduction

Pneumocystis pneumonia (PCP) is an emerging infectious disease in immunocompromised hosts such as those with human immunodeficiency virus (HIV) infection, hematological malignancies, solid tumors, organ transplantations, and connective tissue diseases [1]–[5]. During the past 30 years, the worldwide epidemic of HIV dramatically increased the prevalence of PCP, and it is recognized as one of its most common complications [6]. At present, PCP remains an important cause of HIV-associated pneumonia but the incidence of PCP has decreased largely due to highly active antiretroviral therapy (HAART) and the use of routine prophylaxis against PCP when the CD4 count is <200 cells/µl [7]–[9]. However, the number of patients receiving hematopoietic stem cell and solid organ transplants has increased, and oncologists and rheumatologists have treated patients with progressively more potent immunosuppressive drugs, including high-dose corticosteroids, radiotherapy, tumor necrosis factor-α inhibitors and immunomodulating monoclonal antibodies, which led to an increase in cases of PCP in these populations [10]–[13].

The clinical manifestations are quite different between non-HIV infected PCP (NH-PCP) patients and HIV infected PCP (HIV-PCP) patients, and also vary among patients with different underlying diseases [5], [14]–[16]. Research shows that the clinical outcome of NH-PCP is worse than that of HIV-PCP [17]. The mortality of NH-PCP patients ranges from 30 to 60% [8], compared with 11.3 to 20% in HIV-PCP patients [8], [18], [19]. In addition, in NH-PCP patients who require mechanical ventilation mortality is as high as 60 to 75% [20], [21].

However, few studies have compared the clinical manifestations, treatment and outcome of HIV-PCP and other immunocompromised patients in mainland China. The aim of this retrospective study was to analyze the characteristics of PCP patients in both groups, and to identify clinical factors that contributed to survival.

## Patients and Methods

### Ethics statement

This study was approved by Medical Ethics Committee of Beijing Chaoyang Hospital (NO. 13-ke-80). Due to the retrospective nature of the study, informed written consent was waived, and informed consent was therefore not obtained.

### Study population

Patients discharged with a diagnosis of PCP between 1 January 2008 and 31 December 2012 at two centers of the Capital Medical University (Beijing, China), Beijing Chaoyang Hospital and Beijing Ditan Hospital, both tertiary care teaching hospitals, were reviewed in this study. We adopted the International Classification of Diseases (10th revision) classification codes on a computerized hospital records search system. Patients diagnosed with PCP were screened, and their medical records were carefully reviewed. Data were extracted manually, recorded on study forms and entered into a computerized database.

### Diagnosis of PCP

Because Pneumocystis cannot readily be cultured in the laboratory, the microscopic demonstration of the organisms in respiratory specimens has been the gold standard for the diagnosis of PCP [8]. It is not a sensitive test for diagnosing PCP in non-HIV patients, with previous reports of positive results ranging from 55 to 92% in HIV patients [22], [23] compared with10% in non-HIV patients [23]. It is has been reported that serum β-D-glucan assays have a 92 to 100% sensitivity for definite PCP [24]–[27]. Therefore, this study was based on the following diagnostic criteria:

1. One of the following host factors: receipt of an allogeneic stem cell or solid organ transplant; prolonged use of corticosteroids; treatment with other recognized T cell immunosuppressants during the past 90 days; inherited severe immunodeficiency; chronic underlying disease, or major operations; (2) Clinical criteria: one of the following clinical manifestations such as nonproductive cough, dyspnea on exertion, fever, chest pain, or chills; high-resolution computed tomography (HRCT) typically showed diffuse ground glass opacity (GGO) with patchy distribution; aggressive antibiotic treatment was not valid until the use of PCP-specific treatment; β-D-glucan values of >20 pg/ml (commercial cut-off value), and (3) Mycological criteria: microscopic examination revealed the presence of Pneumocystis cystic or trophic forms in the respiratory samples or the respiratory sample tested positive for Pneumocystis DNA based on the PCR results. Definite PCP was defined as either the presence of Pneumocystis microorganisms in the respiratory samples upon microscopic examination, or the respiratory sample tested positive for Pneumocystis DNA based on PCR results and serum tested positive for G test or a response to anti-PCP treatment. Samples of bronchoalveolar lavage fluid, induced sputum or nasotracheal secretion were routinely investigated for other pulmonary pathogens.

The diagnostic standard for adult HIV/AIDS formulated by the U.S. Centers for Disease Control and Prevention (CDC) in 1993 was used in this study. The antibody test for AIDS used confirmatory Western blot analysis conducted in the local CDC lab to confirm the presence of positive HIV-1 antibodies.

### Data collection

In detail, the collected information included the following parameters: (1) demographic characteristics, (2) medical history: clinical symptoms, therapy and outcome, (3) clinical examination: initial vital signs and lung signs, (4) laboratory data, (5) immune status, and (6) microbiological data: respiratory ducts sample culture.

Lymphopenia was defined as a lymphocyte count lower than 1×10^9^ cells/L. The dosage of steroids was expressed as the prednisone equivalent. Survival data were obtained by telephone contact.

### Statistical analysis

All analyses were performed using SPSS for Windows (version 17). Comparison of two independent samples were performed using independent t tests, χ^2^ tests or continuity correction χ^2^ or Fisher's exact tests, and Wilcoxon rank-sum test, as appropriate. The time-to-event analysis was performed using Kaplan–Meier estimates and the log-rank test. The prognostic analysis was performed using an enter Cox regression model. Potentially relevant clinical predictors, according to the univariate analyses (*p*<0.05), were introduced into the starting model. Variables reaching an alpha-error of less than 0.1 were included in the multivariate model. Statistical tests were two-tailed and a *P* value less than 0.05 denoted a statistically significant difference.

## Results

In our study, 169 admissions fulfilled the diagnostic criteria. Only patients with a first episode of PCP were included, which involved 151 patients: 105 HIV-infected and 46 non-HIV patients. 102 patients were diagnosed by the presence of Pneumocystis cysts in the respiratory samples, and 49 patients were diagnosed by testing positive for Pneumocystis DNA. 84.8% HIV patients were diagnosed with the initial presentation of PCP.

### Clinical Characteristics of the Patients

We compared demographics, clinical characteristics, and underlying diseases of both groups (Table 1). HIV-PCP patients were predominantly men (93.3% vs. 60.9%; *P*<0.001) and younger (37.51±9.41 vs. 54.67±14.40 years; *P*<0.001) compared to NH-PCP patients (mean±SD). Underlying diseases in the NH-PCP group, included transplant (n = 12; hematopoietic stem cell 1, lung 1, liver 1, kidney 9), connective tissue diseases (n = 11; systemic vasculitis 4, dermatomyositis or polymyositis 2, systemic lupus erythematosus 2, Sjogren syndrome 2, antiphospholipid antibody syndrome 1), hematological malignancy (n = 5; non- Hodgkin's lymphoma 4, aplastic anemia 1), solid tumor (n = 4; lung cancer 2, esophageal cancer 2; 3 out of 4 patients received chest irradiation), nephrotic syndrome(n = 5), chronic lung diseases (n = 4; idiopathic pulmonary fibrosis 2, chronic obstructive pulmonary disease 1, asthma 1), and other conditions (n = 5; dermatologic disease 3, diabetes mellitus 1, surgical operation 1). 1 HIV-PCP patient presented with comorbid idiopathic thrombocytopenic purpura.

**Table 1:** Comparison of demographics, clinical characteristics, underlying diseases of NH-PCP and HIV-PCP patients.

Characteristics	NH-PCP (n = 46)	HIV-PCP (n = 105)	*P*-Value
Demographics			
Age, years	54.67±14.40	37.51±9.41	<0.001
Male gender	28 (60.9)	98 (93.3)	<0.001
Patients according to underlying diseases			
Transplant recipients	12 (26.09)	-	
Connective tissue diseases	11 (23.91)	-	
Hematological diseases	5 (10.87)	1 (0.95)	
Malignant hematological diseases	5 (10.87)	-	
Benign hematological diseases	-	1 (0.95)	
Solid tumor	4 (8.70)	-	
Nephrotic syndrome	5 (10.87)	-	
Chronic pulmonary diseases	4 (8.70)	-	
Other conditions	5 (10.87)	-	
Symptomology and clinical presentation		-	
Cough	37 (80.4)	79 (75.2)	0.49
Dyspnea	36 (78.3)	84 (80.0)	0.81
Fever	41 (89.1)	95 (90.5)	0.78
Chest pain	3 (6.5)	14 (13.3)	0.22
Loss of weight	12 (26.1)	65 (61.9)	<0.001
Pneumothorax	2 (4.3)	4 (3.8)	1.00
Laboratory results			
Hgb, g/L	116.00±25.69	119.69±22.02	0.37
Lymphopenia,%	32 (69.6)	71 (67.6)	0.81
TP,g/L	56.63±8.67	72.07±9.54	<0.001
ALB,g/L	26.75±6.20	31.96±5.63	<0.001
LDH,u/L	338.00 (175.00–531.00)	297.80 (188.50–879.00)	0.80
Pct(n = 37/54),ng/ml	0.14 (0.05–17.18)	0.08 (0.05–62.37)	0.71
CRP (n = 34/98),mg/dl	21.62 (0.19–160.00)	53.30 (1.30–260.40)	<0.001
ESR (n = 39/100),mm/h	33.85±19.95	62.92±28.00	<0.001
CD4 T cell count (n = 27/92),cells/µl	182.00 (17.00–847.00)	22.00 (2.00–151.00)	<0.001
CD8 T cell count (n = 27/92),cells/µl	224.00 (29.00–484.00)	411.00 (76.00–1017.00)	<0.001
CD4/CD8 (n = 27/92),%	1.51 (0.1–3.69)	0.05 (0.01–0.38)	<0.001

Data are presented as medians (range) or numbers (%) or mean ± SD, as appropriate. ALB  =  albumin; CRP  =  C-reactive protein; ESR  =  erythrocyte sedimentation rate; Hgb  =  hemoglobin; LDH  =  lactate dehydrogenase; Pct  =  procalcitonin; TP  =  total protein.

Both NH-PCP and HIV-PCP patients had documented reports of cough (80.4% vs. 75.2%, *P* = 0.49), dyspnea (78.3% vs. 80.0%, *P* = 0.81), fever (89.1% vs. 90.5%, *P* = 0.78), chest pain (6.5% vs. 13.3%, *P* = 0.22), and pneumothorax (4.3% vs. 3.8%, *P* = 1.00), respectively. However, significantly fewer NH-PCP patients weight loss (26.1% vs.61.9%, *P*<0.001).

### Use of immunosuppressive therapy and PCP prophylaxis

A total of 42 (91.3%) of the NH-PCP patients were receiving immunosuppressants for their underlying diseases. One HIV-PCP patient was receiving prednisone 10 mg/day for 60 days for idiopathic thrombocytopenic purpura. In the NH-PCP group, glucocorticoid monotherapy was administered in 11 patients (26.2%), otherwise immunosuppressants alone or chemotherapeutic agents alone were administered in 7 patients (16.7%), glucocorticoids combined with immunosuppressive or chemotherapeutic agents were administered in 21 patients (50.0%), radiotherapy alone or combined with chemotherapeutic agents was administered in 3 patients (7.1%).

The time from beginning immunosuppressive medication to PCP diagnosis ranged from 35 to 2920 days, and in 14 patients (33.3%) it was a period of less than 3 months. NH-PCP patients receiving glucocorticoids monotherapy were admitted to the hospital with a daily dose of prednisone equivalent of 25 mg (10–85 mg) for 80 days (35–370 days) [median (range)]. In 13 (31.0%) of the NH-PCP patients treated with glucocorticoids, dosage was either lowered or given as pulse-therapy with sudden discontinuation in the 2 weeks preceding the onset of symptoms. 69.2% of these 13 patients had lymphopenia. Most transplant patients (63.6%, 7/11) were receiving low-dose glucocorticoids during the 30 days before admission.

Time between transplantation and admission for PCP was 180 days (75–1095 days). Patients who had undergone organ or stem cell transplant remained at risk for PCP for many years after transplantation. Only 1 renal transplant patient had received trimethoprim–sulfamethoxazole (TMP-SMX) 160–800 mg twice a week for 3 months for PCP prophylaxis.

### Coinfections

Possible pulmonary coinfections were detected in both NH-PCP and HIV-PCP patients [16 (34.8%) vs. 36 (34.3%), *p* = 0.95], with 15 patients infected by 2 or more pathogens simultaneously. Positive serum assay for pp65 (cytomegalovirus) was identified in 21 patients in both NH-PCP and HIV-PCP groups [5 (7.8%) vs. 16 (15.2%)], EBV-DNA (Epstein-Barr virus) in 4 patients [1 (2.2%) vs. 3 (2.9%)], respectively. Other pathogens found in respiratory samples were Mycobacterium tuberculosis [n = 9, 0 (0.0%) vs. 9 (8.6%)], Pseudomonas aeruginosa [n = 7, 6 (13.0%) vs. 1 (1.0%)], Klebsiella pneumonia [n = 4, 2 (4.3%) vs. 2 (1.9%)], Enterobacter cloacae [n = 4, 1 (2.1%) vs. 3 (2.9%)], Aspergillus [n = 3, 4 (8.7%) vs. 1 (1.0%)], Escherichia coli [n = 3, 2 (4.3%) vs. 1 (1.0%)], Acinetobacter baumannii [n = 2, 1 (2.1%) vs. 1 (1.0%)], Staphylococcus aureus [n = 2, 2 (4.3%) vs. 0 (0.0%)], Klebsiella oxytoca [n = 2, 1 (2.1%) vs. 1 (1.0%)], Cryptococcus neoformans [n = 2, 0 (0.0%) vs. 2 (1.9%)], Stenotrophomonas maltophilia [n = 1, 1 (2.1%) vs. 0 (0.0%)], and H1N1 flu virus [n = 1, 1 (2.1%) vs. 0 (0.0%)], respectively.

### Treatment and outcome

112 patients received TMP-SMX (720 mg of trimethoprim, 3600 mg of sulfamethoxazole daily), 8 patients received caspofungin (50 mg daily) due to TMP-SMX intolerance, and 31 patients received TMP-SMX (720 mg of trimethoprim, 3600 mg of sulfamethoxazole daily) combined with caspofungin (50 mg daily). Adverse effects of TMP-SMX included liver dysfunction (n = 10), minor myelosuppression (n = 9), nausea and/or vomiting (n = 7), rash (n = 3), and minor renal dysfunction (n = 1) were recorded in 30 (19.9%) patients during TMP-SMX treatment. Adverse effects of TMP-SMX were more common in HIV-PCP patients [25 (25.3%) vs. 5 (11.4%), *p* = 0.06], but this difference did not reach statistical significance. 139 patients received appropriate antibiotic therapy according to standard bacterial culture of respiratory samples or empiric antibiotic therapy. 70 patients received systemic corticosteroids as adjunctive therapy.

**Figure 1:**
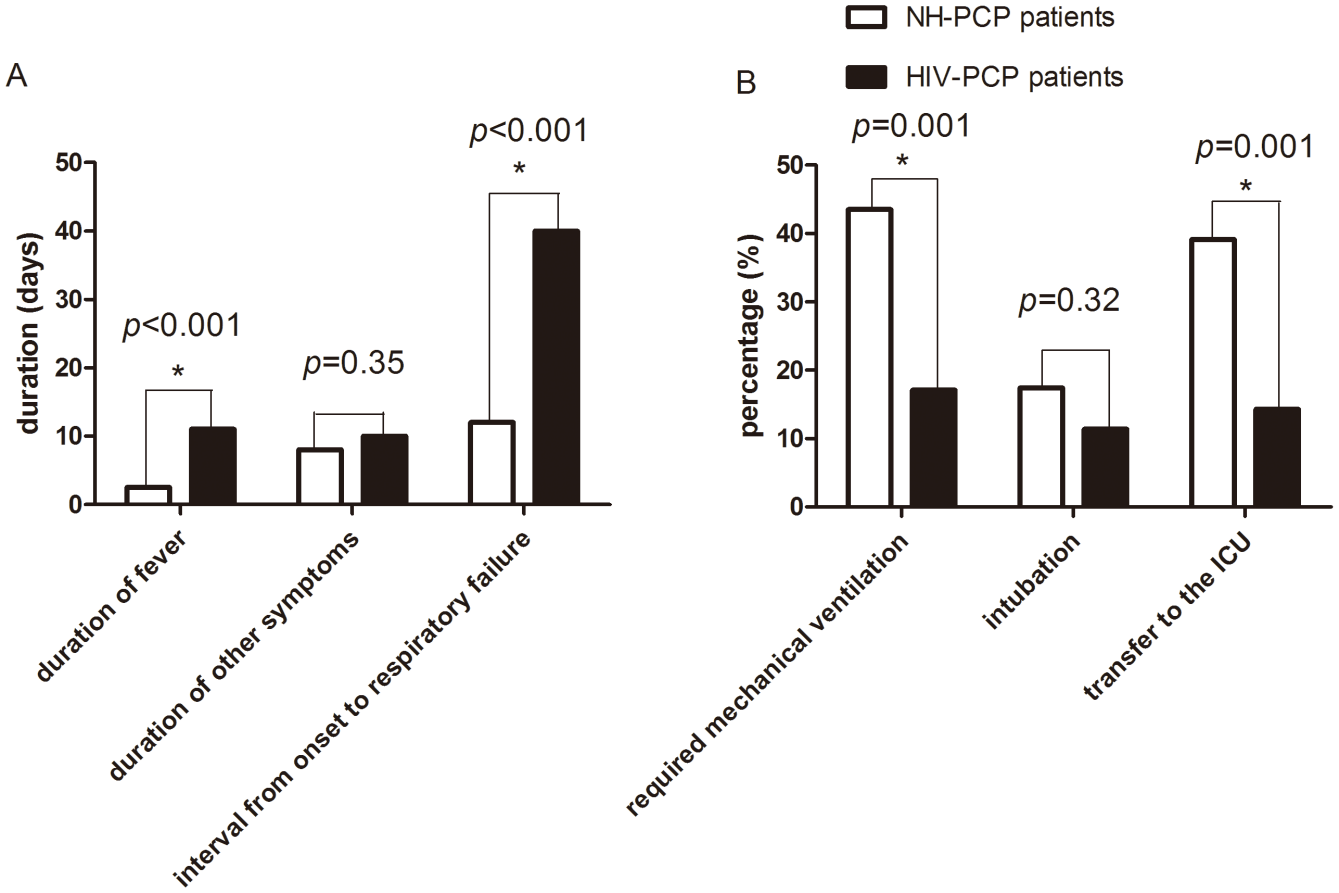
Clinical course. Clinical presentations of the NH-PCP and HIV-PCP patients (A). Secondary endpoints of the two groups (B). The results are reported as median and rate, The comparisons were determined by χ^2^ tests or Wilcoxon rank-sum test. *P<0.05 compared with the corresponding controls.

In survivors in the NH-PCP group, duration of fever [2.5 (1–7) vs. 11 (1–116) days, *p*<0.001] and interval from PCP onset to respiratory failure [12 (5–30) vs. 40 (16–270) days, *p*<0.001] were significantly shorter than survivors in the HIV-PCP group. Duration of other symptoms such as cough and dyspnea were shorter [8 (4–32) vs. 10 (1–88) days, *p* = 0.35], but there was no statistically significant difference (Figure 1).

**Figure 2:**
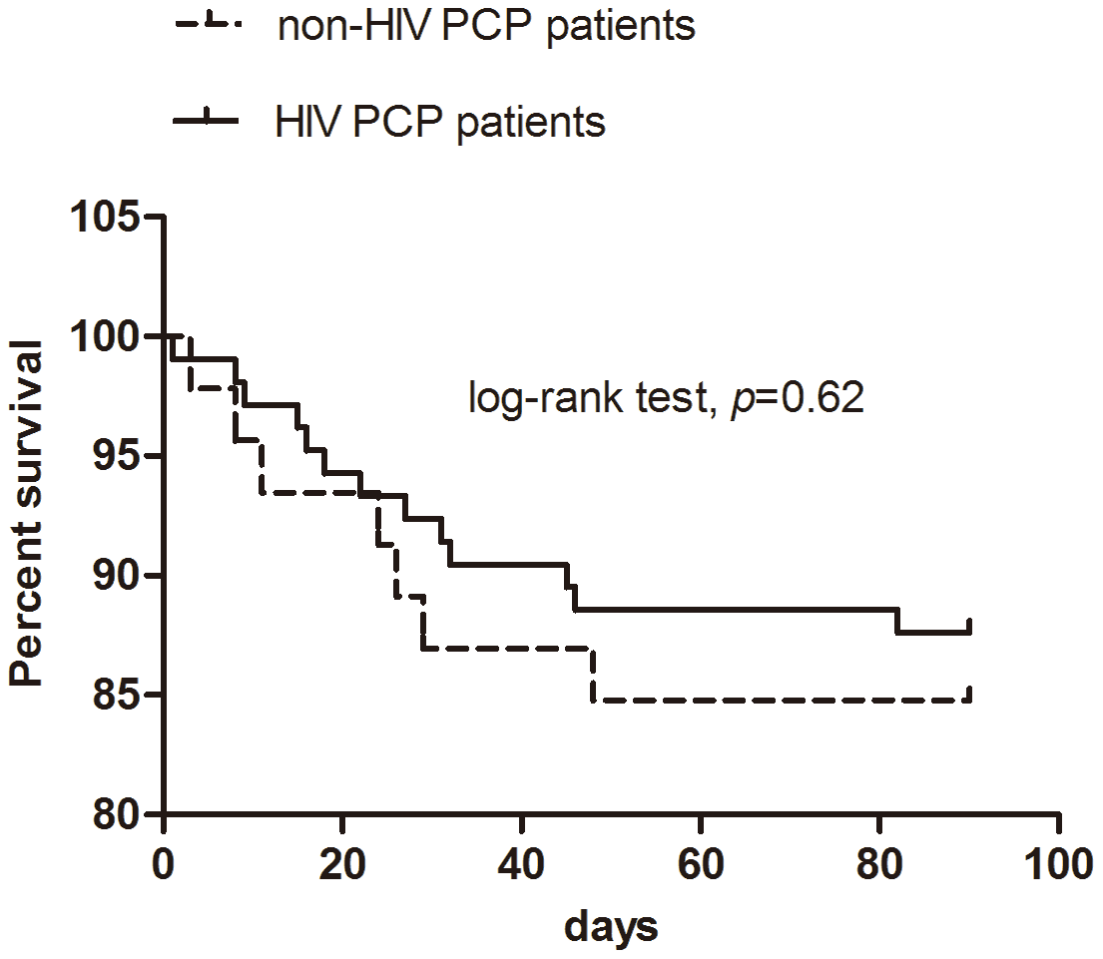
Kaplan–Meier survival curves in patients with pneumocystis pneumonia (PCP) with or without HIV infection.

NH-PCP caused more severe oxygenation impairment (oxygenation index, 285.56±117.86 vs. 324.85±85.74 mmHg, *p* = 0.03), and more patients presenting with acute respiratory failure required mechanical ventilation [20 (43.5%) vs. 18 (17.1%), *p* = 0.001], intubation for ventilatory support [8 (17.4%) vs. 12 (11.4%), *p* = 0.32], and transfer to the ICU [18 (39.1%) vs. 15 (14.3%), *p* = 0.001] (Figure 1). However, the differences for intubation did not reach statistical significance. Moreover, 6 patients refused mechanical ventilation or invasive therapy, and they eventually died. In both NH-PCP and HIV-PCP groups, mechanical ventilation-related mortality [5 (27.8%) vs. 9 (60.0%), *p* = 0.062], all-cause mortality [7 (15.2%) vs. 13 (12.4%), *p* = 0.64], and the results of time-to-event analysis (log-rank test, *p* = 0.62, Figure 2) were similar. One NH-PCP patient with underlying eczema received extracorporeal membrane oxygenation therapy for 8 days on the third day after admission and was discharged after being hospitalized for 21 days.

In the NH-PCP group, transplant recipients accounted for the majority of the reported mortality 3 (42.9%), followed by chronic pulmonary diseases 2 (28.6%), connective tissue diseases 1 (14.3%), and solid tumor 1 (14.3%).

**Figure 3:**
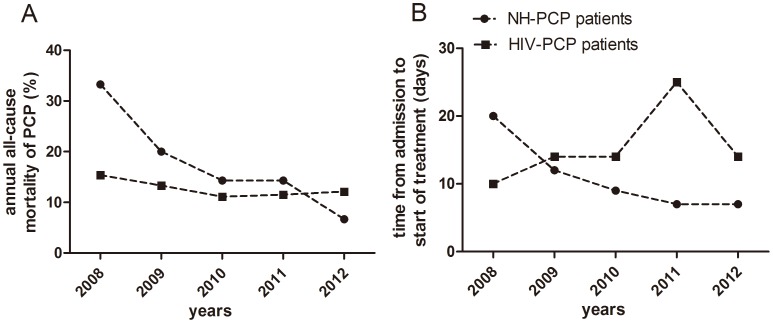
The annual outcome. From 2008 to 2012, all-cause mortality in NH-PCP patients showed a declining trend, but the annual trend of HIV-PCP patients was almost flat. (A).Time from admission to start of treatment in the NH-PCP group showed the same trend as all-cause mortality, but in the HIV-PCP group the trend was not obvious. (B). The results are reported as median and rate.

From 2008 to 2012, time from admission to initial treatment showed a declining trend [20 (9–44) vs. 12 (4–24) vs. 9 (2–23) vs. 7 (2–22) vs. 7 (1–14) days], and all-cause mortality in NH-PCP patients showed the same tendency (33.3% vs. 20.0% vs. 14.3% vs. 14.3% vs. 6.7%). In contrast, in the HIV-PCP group there was no obvious annual trend for time from admission to initial treatment [10 (2–240) vs. 14 (8–210) vs. 14 (4–120) vs. 25 (4–76) vs. 14 (2–55) days] and all-cause mortality (15.4% vs. 13.3% vs. 11.1% vs. 11.5% vs. 12.1%) (Figure 3).

### All-cause mortality after PCP onset

Tables 2 and 3 show the results of univariate and multivariate analyses. In the multivariate analysis, corrections were made for age, gender, four or more clinical manifestations (cough, dyspnea, fever, chest pain, and weight loss), admission to ICU, procalcitonin, and serum albumin. In the NH-PCP group, the presence of any four clinical manifestations [adjusted HR (AHR) 29.06, 95% CI 2.13–396.36, *P* = 0.01], and admission to ICU (AHR 22.55, 95% CI 1.36–375.06, *P* = 0.03) were independent predictors of all cause mortality. In the HIV-PCP group, serum albumin ≤30 g/L (AHR 9.93 95% CI 1.69–58.30, *P* = 0.01), and admission to ICU (AHR 72.26, 95% CI 11.76–443.87, *P*<0.001) were independently associated with all-cause mortality.

**Table 2:** Characteristics associated with mortality in a univariate Cox regression analysis in patients with PCP.

	Unadjusted HR(95% CI)	*P*-value	Unadjusted HR(95% CI)	*P*-value
Age, years	1.03 (0.97–1.09)	0.40	1.04 (0.99–1.09)	0.10
Gender, male	1.22 (0.27–5.43)	0.80	0.05 (0.00–518.81)	0.51
oxygenation index≤300	69.19 (0.16–30395.79)	0.17	4.11 (1.34–12.57)	0.01
Neu%≥85%	6.75 (0.81–56.11)	0.08	7.32 (2.25–23.81)	0.001
Lym%≤10%	62.96 (0.14–27618.67)	0.18	3.88 (1.27–11.86)	0.02
ALB≤25 g/L	2.39 (0.53–10.66)	0.26	2.39 (0.66–8.69)	0.19
ALB≤30 g/L	42.84 (0.07–24998.30)	0.25	3.44 (1.06–11.17)	0.04
Pct≥0.5 ng/ml	9.37 (1.81–48.44)	0.008	19.34 (5.30–70.57)	<0.001
CD4 cell count≤50 cells/µl	-	-	4.89 (0.64–37.58)	0.13
four or more clinical manifestations (cough, dyspnea, fever, chest pain, and weight loss)	14.31 (1.72–119.18)	0.01	1.08 (0.36–3.22)	0.89
Coinfection	13.36 (1.61–111.12)	0.02	0.82 (0.25–2.68)	0.75
Admission to ICU	11.99 (1.44–99.74)	0.02	52.71 (11.55–240.52)	<0.001
Mechanical ventilation	110.98 (0.19–63845.43)	0.15	91.90 (11.88–711.12)	<0.001
Time from admission to initial treatment, days	1.05 (0.99–1.12)	0.08	1.01 (1.00–1.02)	0.02

ALB  =  albumin; CI  =  confidence interval; HR  =  hazard ratio; ICU  =  intensive care unit; Lym  =  lymphocyte; Neu  =  neutrophil; Pct  =  procalcitonin.

**Table 3:** Characteristics associated with mortality in a multivariate Cox regression analyses in PCP patients.

	NH-PCP patients		HIV-PCP patients	
	Adjusted HR (95% CI)	*P*-value	Adjusted HR (95% CI)	*P*-value
Age, year	1.05 (0.97–1.13)	0.22	1.00 (0.94–1.06)	0.95
Gender, female	1.34 (0.15–12.19)	0.80	0.00(0.00)	0.99
four or more clinical manifestations (cough, dyspnea, fever, chest pain, and weight loss)	29.06 (2.13–396.36)	0.01	0.41 (0.10–1.65)	0.21
Admission to ICU	22.55 (1.36–375.06)	0.03	72.26 (11.76–443.87)	<0.001
ALB≤30 g/L	1.43 (0.12–16.97)	0.78	9.93 (1.69–58.30)	0.01
Pct≥0.5 ng/ml	0.77 (0.03–23.85)	0.88	0.24 (0.06–1.06)	0.06

ALB  =  albumin; CI  =  confidence interval; HR  =  hazard ratio; ICU  =  intensive care unit.

## Discussion

PCP remains a leading opportunistic infection in HIV infected patients [6], and the number of PCP cases in those receiving immunosuppressive drugs has increased [10]. However, epidemiological data on PCP are lacking in mainland China. Moreover, there are limited explicit data about whether there are large differences in mortality, the characteristics between the two populations, and clinical factors that contribute to the survival in China. This investigation is a 5-year, retrospective study of 151 definite cases of PCP admitted to two tertiary medical centers.

The main risk factors for PCP are deficiencies in cellular immunity and the use of immunosuppressive agents. Similar to previous reports [1], [4], our patients developed PCP even following the use of glucocorticoids in a low dose or for a short duration. This is consistent with other studies which indicate that a proportion of non-HIV infected patients developed PCP when receiving glucocorticoids as tapering dosage, and most of these cases had lymphopenia [28].

There are guidelines for prophylaxis against PCP for patients with hematological diseases, solid tumors, and recipients of hematopoietic stem cell transplantation and solid organ transplantation [29]–[31]. For NH-PCP patients with other underlying diseases, there is no consensus approach to patient selection for PCP prophylaxis and duration of prophylaxis. Chemoprophylaxis for HIV-PCP is not initiated until there is a measured decline in the peripheral CD4 counts <200 cells/µl [9]. Data suggest that low peripheral blood CD4 counts may also help identify immunocompromised patients at risk for NH-PCP [16]. However, the role of the peripheral CD4 counts in predicting PCP in this population remains to be defined. In our study, CD4 counts for 59% of non-HIV cases were <300 cells/µl, and 63% were <400 cells/µl. However, this number approaches CD4 counts for healthy individuals (500–1500 cells/µl). Despite the underlying risk factors associated with immunosuppressive agents, 97.8% of NH-PCP patients receiving immunosuppressive therapy in our study were not receiving PCP primary prophylaxis before admission.

84.8% of HIV patients were diagnosed at the initial presentation with PCP. It is a concern that the population at high risk of HIV infection are relatively unaware of the risk of PCP, resulting in late presentation with the infection. Lack of recognition may lead to delayed diagnosis, delayed treatment, and potentially higher mortality. In this era of HAART and effective chemoprophylaxis, PCP could probably have been prevented in these patients with appropriate prophylaxis and treatment.

Our observed mortality rate of 15.2% in the non-HIV group is lower than in several studies, in which rates in the order of 30 to 60% have been reported. However, some studies have also reported mortality rates in the range of 7 to 14% [28], [32]. Previous reports confirm that the interval from admission to the start of PCP-specific treatment and diagnosis was significantly shorter in the survivor group than in the non-survivor group [28], [33]. The positive predictive value for survival was >90% when PCP-specific treatment was started within 3 days after admission [33]. Thus it can be seen that early treatment could improve the outcome of PCP. We also observed that time from admission to initial treatment in both groups showed a trend towards statistical significance in the univariate Cox analysis, although it is not an independent risk factor. From 2008 to 2012, all-cause mortality and time from admission to initial treatment of NH-PCP patients both show a declining trend. However, the same tendency was not observed in HIV-PCP patients. Etiological diagnosis is not a sensitive test for diagnosing PCP in non-HIV patients, and some critically ill patients have difficultly tolerating invasive procedures. Therefore, it is essential to conduct a non-invasive and high sensitivity examination. PCR technique has advantages of being sensitive and nonivasive. Although PCR can detect colonization and still produce a false positive result, clinicians should take account of clinical factors (e.g. clinical presentation) and be able to make the correct diagnosis. Time from admission to initial treatment was shown to decrease year by year. Possibly the relatively low mortality observed in the present and other recent studies may be attributed to early diagnosis, which in our study was determined to be a marker of favorable outcome.

Admission to ICU represented a clinical condition of PCP as a later event that may be regarded as being critically and potentially fatally ill. In the current study, patients with PCP presented with nonspecific symptoms such as fever, cough, dyspnea, chest pain, and pneumothorax. The use of four or more clinical manifestations (cough, dyspnea, fever, chest pain, and weight loss) and albumin ≤30 g/L as predictive factors could enable clinicians to recognize the risk of PCP earlier and avoid further deterioration in the patient's condition.

This study has a number of limitations. First, it is a retrospective analysis of a small population. Retrospective studies may be less reliable in terms of the data collected, particularly physical examination data. A prospective study which includes more cases is necessary. Second, it only includes confirmed PCP patients. Some patients would not be enrolled into this study if they were too severe to confirm the diagnosis. Third, the follow up after hospital discharge was for 90 days, which does not allow an accurate prediction of long-term mortality.

## Conclusions

In summary, it should be noted that PCP can occur even with lymphocyte counts or CD4 counts within the normal range. The majority of NH-PCP patients receiving immunosuppressive therapy were not receiving primary prophylaxis. All cause mortality was similar between the NH-PCP and HIV-PCP groups. The use of four or more clinical manifestations (cough, dyspnea, fever, chest pain, and weight loss) for NH-PCP patients and albumin ≤30 g/L for HIV-PCP patients as predictors could allow early recognition of the risk of PCP and avoid further deterioration in the patient's condition. Early diagnosis and treatment may contribute to improved survival in these patient populations.
